# Characterizing atherosclerotic tissues: *in silico* analysis of mechanical properties using intravascular ultrasound and inverse finite element methods

**DOI:** 10.3389/fbioe.2023.1304278

**Published:** 2023-12-13

**Authors:** Álvaro T. Latorre, Miguel A. Martínez, Estefanía Peña

**Affiliations:** ^1^ Aragón Institute for Engineering Research (I3A), University of Zaragoza, Zaragoza, Spain; ^2^ CIBER de Bioingeniería, Biomateriales y Nanomedicina (CIBER-BBN), Zaragoza, Spain

**Keywords:** atherosclerosis, coronary, inverse finite element analysis, material characterization, optimization, vulnerability

## Abstract

Atherosclerosis is a prevalent cause of acute coronary syndromes that consists of lipid deposition inside the artery wall, creating an atherosclerotic plaque. Early detection may prevent the risk of plaque rupture. Nowadays, intravascular ultrasound (IVUS) is the most common medical imaging technology for atherosclerotic plaque detection. It provides an image of the section of the coronary wall and, in combination with new techniques, can estimate the displacement or strain fields. From these magnitudes and by inverse analysis, it is possible to estimate the mechanical properties of the plaque tissues and their stress distribution. In this paper, we presented a methodology based on two approaches to characterize the mechanical properties of atherosclerotic tissues. The first approach estimated the linear behavior under particular pressure. In contrast, the second technique yielded the non-linear hyperelastic material curves for the fibrotic tissues across the complete physiological pressure range. To establish and validate this method, the theoretical framework employed *in silico* models to simulate atherosclerotic plaques and their IVUS data. We analyzed different materials and real geometries with finite element (FE) models. After the segmentation of the fibrotic, calcification, and lipid tissues, an inverse FE analysis was performed to estimate the mechanical response of the tissues. Both approaches employed an optimization process to obtain the mechanical properties by minimizing the error between the radial strains obtained from the simulated IVUS and those achieved in each iteration. The second methodology was successfully applied to five distinct real geometries and four different fibrotic tissues, getting median *R* ^2^ of 0.97 and 0.92, respectively, when comparing the real and estimated behavior curves. In addition, the last technique reduced errors in the estimated plaque strain field by more than 20% during the optimization process, compared to the former approach. The findings enabled the estimation of the stress field over the hyperelastic plaque tissues, providing valuable insights into its risk of rupture.

## 1 Introduction

Atherosclerotic plaques in coronary arteries can trigger diverse acute syndromes, including angina or myocardial infarction (Gutstein and Fuster, 1999). Briefly, atherosclerotic plaques are the result of cholesterol deposition inside the artery walls. That leads to a lipid core surrounded by fibrotic tissue, which, in case of rupture, causes a thrombus due to the contact between lipids and blood. Vulnerable plaques are those which are prone to rupture, therefore the fibrous cap thickness (FCT) that separates the lipid core from the blood is widely used to classify the plaque into stable or vulnerable (Virmani et al., 2005). In literature, FCT smaller than 65 *μm* is considered to be vulnerable (Finet et al., 2004). In addition, other geometrical variables are usually considered to determine the risk of rupture, such as the lipid core area or the degree of stenosis (Cilla et al., 2012; Corti et al., 2022). However, as the plaque rupture is the mechanical failure of the fibrotic tissue, the mechanical properties of the plaque tissues also play a key role in the vulnerability (Ohayon et al., 2008; Akyildiz et al., 2016; Gómez et al., 2019). It has been demonstrated that peak stresses on the fibrotic tissue and stress distributions are correlated with the risk of rupture and its location (Ohayon et al., 2005; Versluis et al., 2006). The stress state of the arterial wall could be only accurately calculated by knowing the mechanical behavior of the tissues. The clinical detection and characterization of atherosclerotic plaques remain a challenge for early diagnosis. Nowadays, Intravascular Ultrasound (IVUS) images are one of the most common imaging techniques for the diagnosis of atherosclerotic plaques in coronary arteries.

The mechanical characterization of atherosclerotic tissues is highly dependent on previous tissue segmentation that could be performed manually on IVUS images due to the different echo reflectivity characteristics of the tissues (Olender et al., 2020), using virtual histologies (Kubo et al., 2011) or new methodologies based on machine learning (Sofian et al., 2019; Du et al., 2022). Some studies employed an optimization process to simultaneously segment and obtain the elasticity map of the arterial wall (Le Floc’h et al., 2009; Le Floc’h et al., 2012; Tacheau et al., 2016). In a different approach, Narayanan et al. (2021) achieved segmentation using deep learning techniques on OCT images. These approaches showed how important it is to obtain accurate segmentation results. The mechanical characterization of the properties usually involves three steps. It commonly begins with the acquisition of, at least, two clinical images (base and target shapes), normally in systolic and diastolic pressure (Liu et al., 2019), and then the relative displacements or deformation between them are computed (Le Floc’h et al., 2009; Tacheau et al., 2016; Torun et al., 2022). Secondly, the segmentation of the tissues is performed by using Magnetic Resonance Imaging (MRI), *ex-vivo* testing and histologies (Akyildiz et al., 2016; Torun et al., 2022), optical coherence tomography (OCT) (Narayanan et al., 2021) or segmentation based on mechanical properties (Le Floc’h et al., 2009; Nayak et al., 2017). Thirdly, the last step consists of estimating the mechanical properties by means of an optimization process, where the displacements/strains estimated in the first step are compared with those computed by an inverse finite element analysis (Le Floc’h et al., 2009; Torun et al., 2022). Other approaches tried to match meshes between the base and the target shapes (Liu et al., 2019) or were based on micro-morphological information, like the interfaces of the plaque tissues, to recover the material behavior (Narayanan et al., 2021), and others used the virtual fields method to obtain the material parameters (Avril et al., 2004; Avril et al., 2010). The optimization algorithm plays a key role in determining the mechanical properties, and the choice of algorithm depends on the type of problem we want to solve. The computational cost and the complexity of the process vary greatly depending on the application. To obtain the linear elastic properties of the tissues, a gradient-based optimization procedure could provide robust results (Le Floc’h et al., 2009; Le Floc’h et al., 2010; Tacheau et al., 2016; Porée et al., 2017). However, for more complex material properties, this type of algorithm could become stuck in local minima. Genetic algorithms, such as the Non-dominated Sorting Genetic Algorithm used by Narayanan et al. (2021), select an initial population of parameters, and then propagate the population over several generations. This kind of algorithm allows the evaluation of a large number of material parameters, but it requires a lot of time and computational cost. Nowadays, more complex new optimization methods have emerged that enable the evaluation of complex material models to be evaluated by using machine learning methods, such as the Bayesian optimization (Torun and Swaminathan, 2019; Torun et al., 2022). A very different approach, like the principal component analysis optimization used by Liu et al. (2019), permits optimization times of 1–2 h by partitioning the possible stress-stretch curves using a dimensional reduction technique (Liu et al., 2018; Liu et al., 2019). As part of the optimization process, several studies used arterial images of two pressure steps within a pressure increment of 5 mmHg between them (Le Floc’h et al., 2009; Nayak et al., 2017). Despite the hyperelastic behavior of the arterial tissues, this procedure allows the application of small deformation theory to estimate the linear elastic properties of the tissues. In these cases, the estimated Young’s modulus (Le Floc’h et al., 2009; Le Floc’h et al., 2010; Le Floc’h et al., 2012; Nayak et al., 2017) or orthotropic modulus (Gómez et al., 2019) refers to the associated relative stiffness at that pressure. Akyildiz et al. (2016) proposed a framework to describe the mechanical properties of atherosclerotic tissues from *ex-vivo* testing images. They estimated the Neo Hookean material parameters for different pressure increments, showing a correlation between increased pressure and increased stiffness. This trend corresponded to the hyperelastic behavior of arterial tissues, with the stress-stretch curve exhibiting greater stiffness at higher loads. In spite of the methodology providing a hyperelastic behavior of the tissues, Neo Hookean parameter values exhibited variations with changes in pressure and failed to describe the high non-linear behavior of the atherosclerotic tissues. To accurately predict the hyperelastic mechanical behavior of tissues, a non-linear analysis using unpressurized geometry should be conducted. IVUS images are taken at a certain pressure, and if these images are assumed to be in an unpressurized configuration, incorrect strain and stress distributions would be obtained. To account for unpressurized geometries, some studies utilized histologies (Akyildiz et al., 2016), *ex-vivo* MRI (Torun et al., 2022) or assumed the first clinical image as stress-free geometry (Narayanan et al., 2021) to obtain the hyperelastic parameters of hyperelastic multi-parameter materials on atherosclerotic carotid arteries.

In this article, we present a theoretical framework to estimate the non-linear mechanical properties of atherosclerotic plaques in coronary arteries based on clinical images. We previously proposed a method based on two consecutive images taken by IVUS for segmenting the different atherosclerotic tissues (Latorre et al., 2022). In addition, in that contribution, we also defined the strategy to simulate the IVUS data from finite element (FE) models. That segmentation enabled us to describe geometrical measures related to plaque vulnerability, such as the FCT or the lipid core area. After the image segmentation, in this paper, we propose to use an inverse FE analysis in order to obtain the mechanical properties of the segmented materials. Since it is an *in silico* study, all the IVUS data are simulated using FE models with some noise over the strain distribution. We introduce two different approaches for estimating the mechanical properties of atherosclerotic tissues. Both of them use the information from two different pressure steps to collect the relative radial strains. In the first method, we determine the linear elastic properties of the tissues through a simple optimization process using those radial strains (Le Floc’h et al., 2009; Bouvier et al., 2013; Tacheau et al., 2016). However, it must be said that this approach only provides the relative stiffness of the tissues at a certain blood pressure. Then, in the second approximation, we implement a process to estimate the non-linear properties of the atherosclerotic tissues. The arterial behavior exhibits high non-linearity, therefore, we include a Pull-Back algorithm to estimate the unpressurized geometry inside the optimization process. This implementation enables us to obtain the hyperelastic properties of plaque materials and an estimated zero-pressure (ZP) geometry. It is worth highlighting that these variables are critical to a proper determination of the stresses on the plaque. At the end of this optimization process, we could evaluate the stress state of the arterial tissue at physiological pressures and evaluate the risk of rupture.

## 2 Materials and methods

The appearance of atherosclerotic tissues on IVUS images varied due to their different echo reflectivity characteristics (Olender et al., 2020). While it was feasible to differentiate calcifications and softer inclusions like lipids through visual inspection, it was not possible to obtain a proper segmentation or estimate mechanical behavior. The aim of this paper was to determine the mechanical properties of atherosclerotic tissues. For this purpose, we compared two different methods for determining the mechanical properties as linear elastic or non-linear hyperelastic. The first one estimated the linear elastic properties of the tissues by applying incremental pressure. This resulted in a measurement of the relative stiffness of the tissues at a specific pressure. While this method allowed for the quantification of the relative modulus of elasticity of atherosclerotic tissues, it did not enable the determination of the stress state of the plaque throughout the cardiac cycle. It is a common methodology found in literature, where arterial tissues were considered with linear or orthotropic materials (Le Floc’h et al., 2009; Gómez et al., 2019). To overcome this limitation, the second approach included a Pull-Back algorithm in the inverse FE analysis in order to estimate the non-linear properties of the tissues. The use of this algorithm enables the mechanical response of tissues to be analyzed from the unpressurized configuration.

### 2.1 Determination of linear elastic properties

We initially simulated the IVUS data from FE models using Neo Hookean materials. Then an image segmentation was performed to finally obtain the linear elastic properties through an optimization process.

#### 2.1.1 Simulated IVUS data

The IVUS data were simulated using FE models with five real patient IVUS geometries obtained from the literature (Finet et al., 2004; Le Floc’h et al., 2009; Bouvier et al., 2013). IVUS images typically do not enable detection of the adventitia or media layers, this is why only the fibrotic tissue, the lipid core, and calcification were considered. Moreover, clinical images display only the cross-section of the arterial wall, so the FE models were 2D including the plane strain assumption. Since IVUS images were taken under specific pressure, the unpressurized geometry was previously estimated. The robustness of this approach was tested with five geometries and different material combinations of lipid and fibrotic tissues to ensure that the results were consistent regardless of the geometry or material properties. In previous work, Caballero et al. (2023) conducted a study on the elastic modulus ranges for lipid and fibrotic tissues through the literature. They collected a range of [1–100 kPa] for lipid elastic modulus and [390–1,200 kPa] for fibrotic elastic modulus. To cover the whole range of combinations, we performed a Latin hypercube sampling (LHS) to take 15 representative samples (Corti et al., 2020). In this approximation, lipidic and fibrotic tissues were modeled as quasi-incompressible Neo Hooke materials using Eq. 1. Both Eqs 2, 3 establish a relationship between Neo Hookean and linear elastic parameters. The influence of geometry was analyzed with the material properties reflected in Table 1 (Le Floc’h et al., 2009; Babaniamansour et al., 2020), whereas the influence of the material was solely conducted using the first plaque geometry with 15 material combinations obtained from the LHS. In both analyses, calcifications were fixed and modeled as linear elastic material with 5,000 kPa of Young’s modulus and *ν* = 0.333 (Le Floc’h et al., 2009).

$$\Psi =C_{10}\left(I_{1}-3\right)+D_{1}{\left(J-1\right)}^{2},$$ (1)

$$C_{10}=\frac{E}{4\left(1+\nu \right)},$$ (2)

$$D_{1}=\frac{6\left(1-2\nu \right)}{E},$$ (3)

**TABLE 1:** Material properties used for geometrical analysis in the first approach.

	Neo Hooke parameters		Linear parameters	
Tissue	*C*_10_ [*kPa*]	*D*_1_ [*kPa* ^−1^]	*E* [*kPa*]	*ν* [-]
Fibrotic	103.45	0.001	—	
Lipidic	1.72	0.06	—	
Calcification	—		5,000	0.333

**FIGURE 1:**
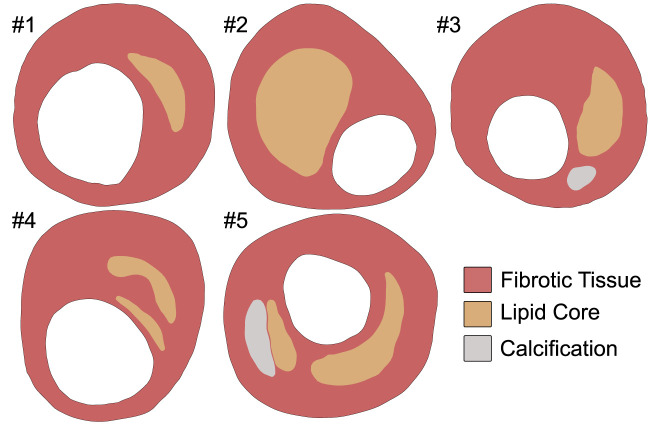
Five real geometries considered in the analysis (Finet et al., 2004).

The analysis was performed in the commercial software *Abaqus* (*Dassault Systems 2014*), where we applied an internal pressure of 115 mmHg in the lumen, which represents the average pressure in patients with high-normal pressure and grade 1 hypertension (Ramzy, 2019). It should be noted that all the geometries were meshed using plain strain three-node linear elements (CPE3). The mesh size was set to achieve at least three elements between the lumen and the lipid core, taking into account the accuracy of the IVUS technique. The five different FE models had a number of elements of 4,375, 6,392, 7,945, 3,173, and 4,207, respectively. Rigid body motion was constrained by fixing three external contour points of the fibrotic tissue (Cilla et al., 2012). Figure 1 shows the five different FE models with their tissues. The FE models simulated the atherosclerotic plaque; to mimic the acquisition of two consecutive IVUS images we used the FE results at various pressure steps. Nowadays, there are several different approaches for estimating displacement or strain fields from two ultrasound images (Maurice et al., 2004). To replicate this, we gathered the nodal coordinates (*X* and *Y*) and displacements (*u*_*x*_ and *u*_*y*_) at pressures of 110 and 115 mmHg. Then, we computed the relative displacements between both pressure steps. This process aimed to simulate the data obtained through displacement estimators on two IVUS images with 5 mmHg between both (Porée et al., 2017). Once we had obtained the relative displacement from the small pressure increment, we were able to calculate the strains under the infinitesimal strain theory. Finally, we added a signal-to-noise ratio (SNR) of 20 dB to the strain fields in order to simulate the intrinsic noise present in IVUS data (Porée et al., 2015). We computed the strains in both Cartesian and cylindrical coordinates, as well as the principal and equivalent strains. However, to be consistent with prior studies (Le Floc’h et al., 2009; Le Floc’h et al., 2012; Tacheau et al., 2016), we mainly utilized radial strains for the segmentation and optimization process due to their lower estimation error from IVUS images compared to other deformation variables.

#### 2.1.2 Segmentation

The segmentation process was fully described previously in Latorre et al. (2022). Briefly, the method was based on the representation of Strain Gradient Variables (SGV). This type of variable highlighted the contours of the different atherosclerotic tissues and after a Watershed-Gradient Vector Flow segmentation it was possible to extract the plaque components. The segmentation results varied depending on the chosen SGV; in this work, we segmented all the lipids and calcifications with the modulus of the gradient of the radial strains $\left(\left|▿\varepsilon_{rr}\right|\right)$ alone or in combination with other SGVs like ( $\left|▿\varepsilon_{rr}\right|+\left|▿\varepsilon_{\min}\right|$ , $\left|▿\varepsilon_{rr}\right|+\left|▿\varepsilon_{\mathrm{vMises}}\right|$ …). We also proved the accuracy of the method by measuring FCT and lipid areas in some geometries. The segmentation was performed through imaging techniques, so we converted the extracted tissues from images to meshes. We used the Partial Differential Equation toolbox from *Matlab* (*version R2022b*, Mathworks, MA, United States) to build the FE model from the segmented tissues. As a result of this process, we obtained a segmented FE model of the plaque at 110 mmHg.

#### 2.1.3 Mechanical characterization

**FIGURE 2:**
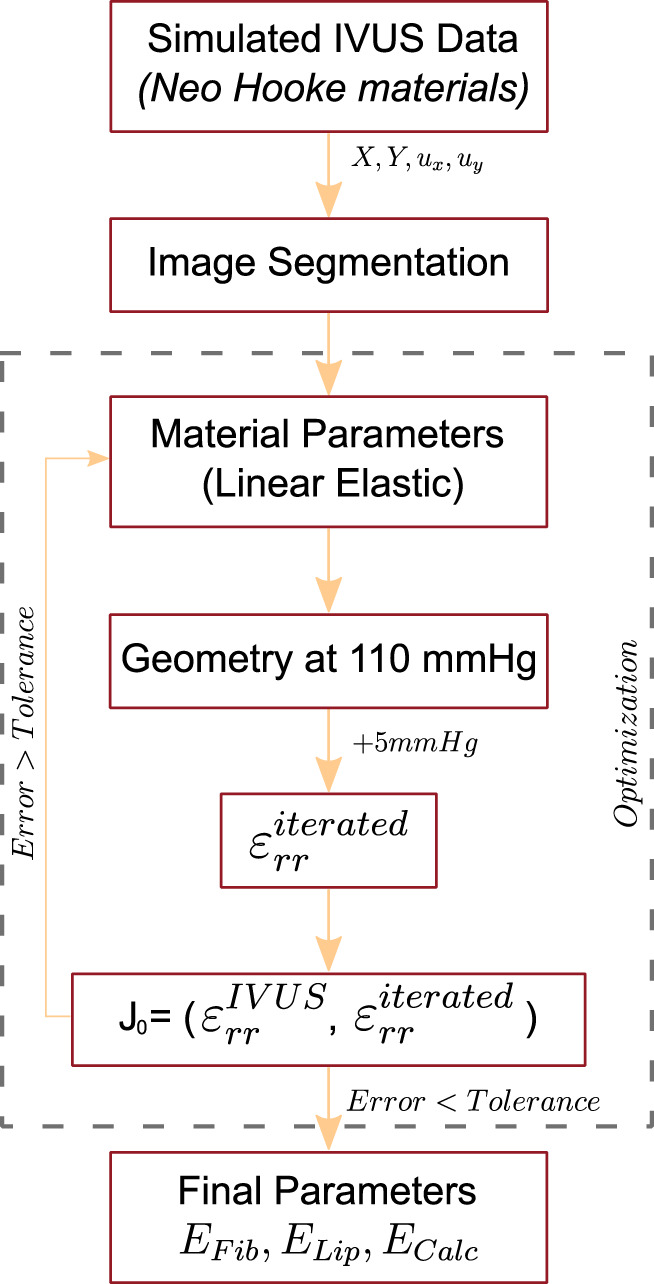
Scheme of the optimization process of the first method to recover the linear elastic properties of the atherosclerotic tissues.

After the segmentation at 110 mmHg, the estimated radial strains $\left(\varepsilon_{rr}^{\mathrm{iterated}}\right)$ were computed by imposing a lumen pressure of 5 mmHg and optimizing the material properties of the segmented tissues. Since the pressure increment happens to be low, we assumed a linear elastic behavior of the materials (Le Floc’h et al., 2009; Tacheau et al., 2016). In addition, lipid core and fibrotic tissues were considered quasi-incompressible materials with a fixed Poisson’s ratio of 0.49, while calcifications were considered an isotropic material with a fixed Poisson’s ratio of 0.33 (Babaniamansour et al., 2020; Corti et al., 2020). Therefore, only the elastic modulus of the fibrotic tissues, lipid, and calcification (*E*_*Fib*_, *E*_*Lip*_ and *E*_*Calc*_) varied during the optimization. The optimization was performed by linking *Matlab* and *Abaqus* software (Papazafeiropoulos et al., 2017) and also using a pattern-search algorithm, which works with smooth and non-smooth functions as it is not based on gradient descent. This optimization algorithm partitions the space of the objective function into mesh points and starts evaluating the function at an initial point. Then, it uses that information to generate additional points to find the parameters that most minimize the cost function (Hooke and Jeeves, 1961). At each iteration, the algorithm polled different mesh points and if the method finds a point that minimizes the function, the polling mesh size will increase by two; otherwise, the polling mesh size will decrease by half. The selected poll algorithm was the Generating Set Search which is more efficient for linear-constrained problems than the classic algorithm. The target cost function (*J*_0_) to minimize was the Normalized-Root-Mean-Squared Error (NRMSE) between the simulated IVUS radial strains $\left(\varepsilon_{rr}^{\mathrm{IV US}}\right)$ and the estimated by the method $\left(\varepsilon_{rr}^{\mathrm{iterated}}\right)$ shown in Eq. 4. In order to validate this method, we compared the resulting elastic modulus with the Neo Hookean parameters that were employed in the simulated data using Eqs 2, 3. This first approach is schematized in Figure 2.

$$J_{0}\left(\varepsilon_{rr}^{\mathrm{IV US}},\varepsilon_{rr}^{\mathrm{iterated}}\right)=100\sqrt{\frac{\frac{1}{N}\sum {\left(\overset{\mathrm{IV US}}{\underset{rr}{\varepsilon }}-\varepsilon_{rr}^{\mathrm{iterated}}\right)}^{2}}{\left|mean\left(\varepsilon_{rr}^{\mathrm{IV US}}\right)\right|}},$$ (4)

Finally, to evaluate the accuracy of the estimation of the Young’s modulus, we introduced the *Success Rate* (*sr*) coefficient, which quantifies how closely our predicted Young’s modulus (*E*_*estimated*_) matched the actual FE values (*E*_*real*_) Eq. 5.

$$sr\left(\%\right)=100\left(1-\frac{\left|E_{\mathrm{real}}-E_{\mathrm{estimated}}\right|}{E_{\mathrm{real}}}\right),$$ (5)

### 2.2 Determination of non-linear properties

This approach attempted to characterize the mechanical properties of the atherosclerotic tissues as hyperelastic and wanted to provide an estimation of the unpressurized plaque geometry.

#### 2.2.1 Simulated data

We proposed a similar methodology as in the previous approach, with the major difference being the material used for the FE models and the simulation of the ZP geometry. To consider a non-linear hyperelastic material, it is necessary to know the unpressurized configuration where the non-linear stress-strain curve begins. Normally, healthy arterial tissues were assumed to be anisotropic such as the media or adventitia. However, diseased tissues, such as fibrotic or lipid tissues, were considered isotropic exponential-type materials. While lipids were treated as Neo Hookean material, fibrotic tissue was modeled with the Gasser-Ogden-Holzapfel (GOH) strain energy function (Eq. 6) (Gasser et al., 2006). The parameter *D* was fixed to 0.005 to reproduce the quasi-incompressibility behavior of the tissues. Meanwhile, *C*_10_ represented the initial stiffness of the tissue at zero-pressure, and *k*_1_ and *k*_2_ indicated the stiffness at higher pressures and the shape of the exponential curve, respectively. Finally, *κ* was set at 0.3333 to consider an isotropic fiber response. In order to understand the influence of the geometry and composition of the plaque, we analyzed five real geometries and four different fibrotic tissues (cellular, hypocellular, and two calcified) (Loree et al., 1994; Versluis et al., 2006). The material parameters for the GOH model are presented in Table 2 where the “Calcified 1” material was the calcified fibrotic tissues used in the different geometrical analyses. Moreover, atherosclerotic plaques could present calcifications as highly rigid inclusions. These calcifications were considered isotropic linear elastic materials with *E* = 5,000 *kPa* and *ν* = 0.333.

$$\Psi =\frac{1}{D}\cdot {\left[J-1\right]}^{2}+\mu \left[I_{1}-3\right]+\frac{k_{1}}{2k_{2}}\underset{i=4,6}{\sum }\left(exp\left(k_{2}{\left[\kappa \left[I_{1}-3\right]+\left[1-3\kappa \right]\left[I_{i}-1\right]\right]}^{2}\right)-1\right),$$ (6)

**TABLE 2:** GOH material parameters used for the simulated fibrotic tissues.

	*C*_10_ [*kPa*]	*k*_1_ [*kPa*]	*k*_2_ [−]
Calcified 1	9.58	17,564	0.51
Calcified 2	17.29	13968.82	3.36
Cellular	0.1	1948.80	3.36
Hypocellular	139.32	15918	0.1

Finally, the displacement or strain fields obtained from two consecutive IVUS images and the image segmentation process were simulated following the same guidelines defined in the former approach.

#### 2.2.2 Optimization methodology

**FIGURE 3:**
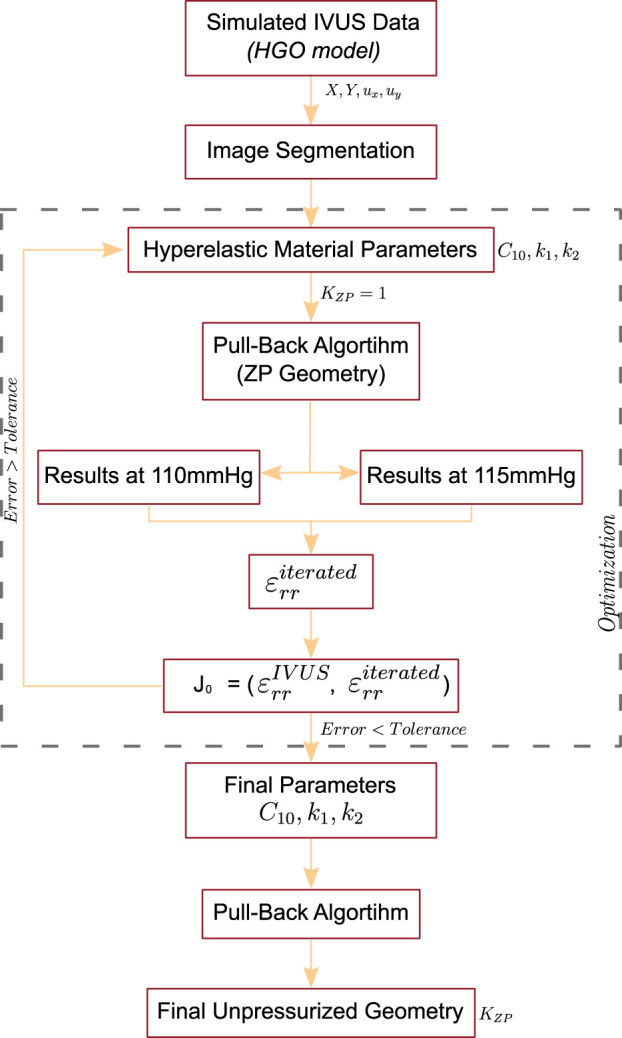
Scheme of the optimization process of the second method to recover the non-linear hyperelastic properties of the atherosclerotic tissues.

We developed a novel pipeline to characterize the non-linear properties of the plaque tissues. After segmentation (see Section 2.1.2), an optimization process was conducted to obtain the hyperelastic mechanical properties of the tissues. During each iteration, the methodology involved three different steps. First, the optimization algorithm selected an initial seed to iterate the material parameters and generated the initial FE model. Then, a Pull-Back algorithm was employed to estimate the ZP geometry. Secondly, it collected the radial strain within the pressures of 110 and 115 mmHg $\left(\varepsilon_{rr}^{\mathrm{iterated}}\right)$ . The objective of the algorithm was to minimize the cost function error between the simulated IVUS strains and the strains obtained during the iteration (Eq. 4). These two steps were repeated until the resulting error was less than a tolerance of 10^–4^ or the optimization time exceeded 4 hours. Finally, we estimated the unpressurized geometry with the final material parameters. The entire process is described below and is illustrated in Figure 3.

• **First Step**. On each analysis, only three material parameters needed to be optimized for the fibrotic tissue (*C*_10_, *k*_1_ and *k*_2_ for isotropic GOH materials), two for each lipid (*C*_10_ and *D*_1_ for Neo Hooke materials), and one for each calcification (*E* for linear elastic materials). To recover an approximated ZP geometry we used an adapted version of the Pull-Back algorithm developed by Raghavan et al. (2006). This algorithm was originally created to obtain the ZP geometry of a 3D arterial aneurysm, so it was modified to recover the initial geometry of 2D atherosclerotic plaques. The algorithm created an initial FE model with the segmented pressurized geometry and added an internal pressure of 110 mmHg (the pressure at which the segmentation was performed). Then, the resulting nodal displacements $\left(\left[u_{x}^{ZP}u_{y}^{ZP}\right]\right)$ were collected. Unpressurized geometry was obtained by constructing a new FE model using the pressurized segmented geometry and imposing the nodal displacements multiplied by a recovery factor (*K*_*ZP*_) as a boundary condition (BC), as depicted in Eq. 7. The final ZP geometry was achieved through an iterative process by varying the recovery factor and comparing the error of the coordinates between the ZP geometry candidate, after adding 110 mmHg, and the pressurized segmented geometry. Raghavan et al. (2006) considered the recovery factor as a parameter that should be optimized. However, we assumed a recovery factor *K*_*ZP*_ = 1, which was an intermediate value, with the aim of avoiding another optimization process and also reducing the computational cost.

$$BC=-K_{ZP}\left[\begin{array}{c} u_{x}^{ZP}\\ u_{y}^{ZP} \end{array}\right]$$ (7)

**FIGURE 4:**
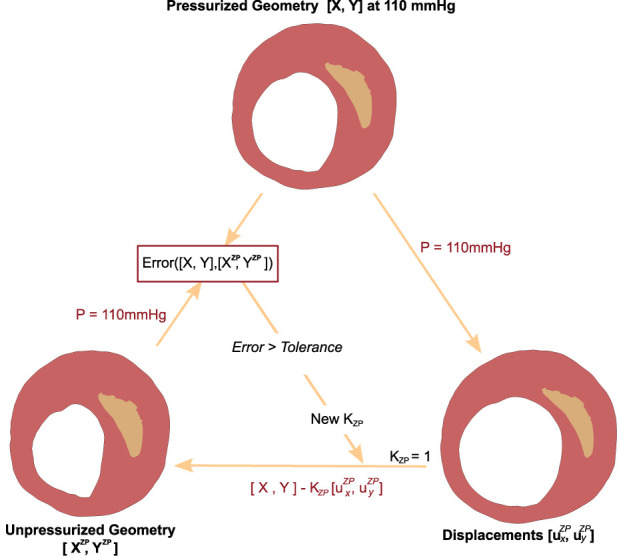
Scheme of the Pull-Back algorithm used to recover the Zero-Pressure geometry.

• **Second Step**. Once the unpressurized geometry was obtained, we imposed an internal pressure of 115 mmHg at this unpressurized geometry to compute the iterated radial strain $\left(\varepsilon_{rr}^{\mathrm{iterated}}\right)$ . At this stage, we used the same cost function defined in Eq. 4 and the same pattern-search optimization algorithm (see Section 2.1.3). In linear elastic materials, as in the previous approach, due to its simplicity, the initial size of the polling mesh was equal to 1. However, GOH materials are more complex which could lead to a higher number of local minima in the cost function. Thus, we changed the initial mesh size of the poll algorithm to 100 to cover more different variable possibilities and also to avoid being stuck in local minima. Hence, after analyzing the initial point, the algorithm evaluated different mesh points with a distance of 100 between them, allowing many different parameter combinations to be analyzed. Furthermore, the optimization process needed a search range for each material parameter, and the pattern-search algorithm used this range to give more relevance to those variables with a greater search range. So, the ranges for fibrotic GOH parameters were $C_{10}\left[1\to 50\;kPa\right]$ , $k_{1}\left[5\to 100000\;kPa\right]$ , $k_{2}\left[1\to 100\right]$ obtained from curve fitting of literature data (Loree et al., 1994; Versluis et al., 2006), and giving more relevance to *k*_1_ which proved to be more important in the mechanical response at physiological pressures. Both the lipid and the calcification ranges were adjusted considering the proposed range of Caballero et al. (2023), with the lipid parameters $C_{10}\left[0.1\to 100\;kPa\right]$ and $D_{1}\left[0.005\to 0.9\;kPa^{-1}\right]$ and the elastic modulus of the calcification with a range of $\left[500\to 10000\;kPa\right]$ .
• **Third Step.** After completing the optimization process, we obtained the hyperelastic properties of the tissues, however, the resulting unpressurized geometry was obtained with the recovery factor *K*_*ZP*_ fixed to 1. In this final step, we implemented the whole Pull-Back algorithm, optimizing the value of *K*_*ZP*_. The process was the same as described in the first step, with the difference of changing the recovery value. Figure 4 shows a scheme of the iterative Pull-Back process. This method was implemented into the five different geometries and the four distinct fibrotic materials (cellular, hypo-cellular, and two calcified).

## 3 Results

The proposed segmentation methodology was previously presented and validated (Latorre et al., 2022), so no comments about that have been included here. Regarding the mechanical characterization, we present the results of both approaches.

### 3.1 Determination of linear elastic properties

In the first approach, we obtained the relative Young’s modulus of the tissues at 110–115 mmHg of blood pressure. In order to validate the approach, five geometries and fifteen material combinations of lipid-fibrotic tissues were analyzed using FE models with Neo Hookean materials. We computed the (*sr*) coefficient for the different cases.

**FIGURE 5:**
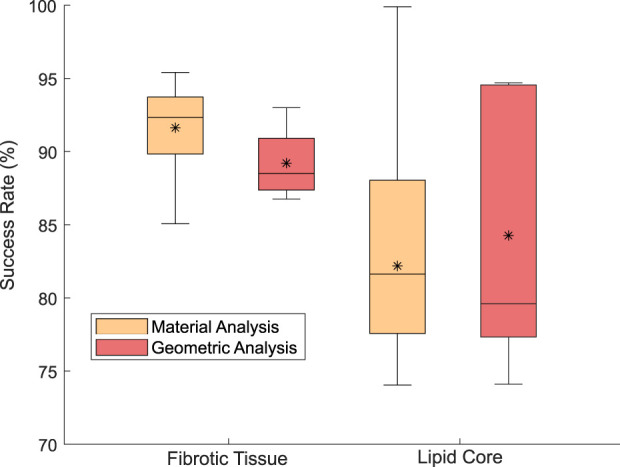
Box plot of the *sr* variability in the fibrotic material (left) and lipidic material (right) for the different material combinations of LHS (orange) and different geometries (red).

In Figure 5 there is a box plot of the *sr* for the different geometries and material combinations of the LHS. The FE models of the simulated data used for analyzing the influence of the geometry were constructed with Neo Hooke parameters presented in Table 1, which correspond, using Eqs 3, 4, to Young’s modulus of 600 kPa for the fibrotic tissue and 10 kPa for the lipid (Le Floc’h et al., 2009). The resulting mean elasticity modulus obtained was 535.25 and 10.05 kPa for the fibrotic and lipid core respectively, while the median *sr* for the fibrotic tissue was 88.5% and 79.6% for the lipids core, as we can see in reddish color in Figure 5. The interquartile range for lipid’s *sr* was 4.8 times higher than the fibrotic tissue.

**FIGURE 6:**
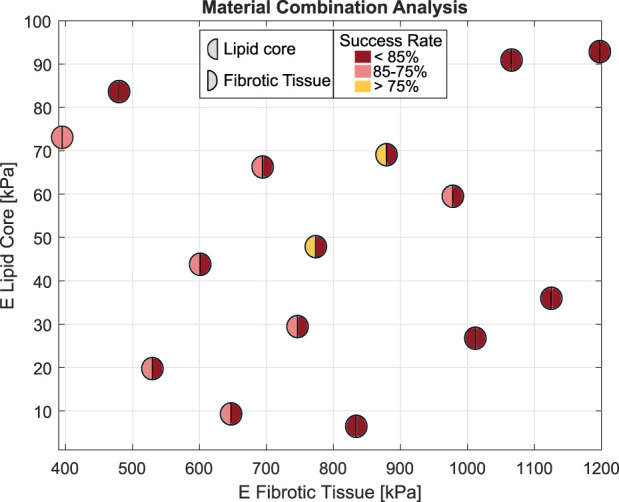
LHS with the differences material combinations between fibrotic-lipid elastic modulus. The left half of the circle presents the *sr* of the lipid core characterization and the right half for *sr* in fibrotic tissues. The colors range from yellow to dark red depending on how high the success rate is.

On the other hand, analyzing the influence of material combinations, orange box plots of Figure 5 show a median *sr* value of 92.33% for fibrotic tissues and 81.62% for lipids. Once again, the interquartile range for lipid *sr* was higher than fibrotic’s. For calcifications, the method detected a highly rigid material with Young’s modulus over 5,000 kPa. However, this did not affect the estimated radial strains used in the cost function. Figure 6 presents the LHS distribution of the lipid-fibrotic material combination, with each data point marked in a different color based on its estimation results. Each circle was divided into two halves: The color of the left half of the circle represents the *sr* of the fibrotic tissue, whereas the color of the right half shows the *sr* of the lipids. The best results were obtained for combinations with higher stiffness in the fibrotic tissue. In contrast, combinations with lower Young’s modulus resulted in a worse *sr* regardless of the stiffness of the lipid tissue. As the linear materials calculations converged quickly, the convergence tolerance was the stopping criterion rather than limiting the optimization time. Each optimization process took about 2–3 h, equivalent to around 180 material evaluations, depending on the complexity of the geometry.

Previously to determine the non-linear properties, we checked the methodology of the first approach using simulated IVUS data from FE models with GOH material model instead of Neo Hookeans. With this test, we checked the availability of the proposed linear methodology to reproduce the response of non-linear tissues. Unlike Neo Hooke materials, GOH parameters did not have a direct relationship with Young’s modulus, so a direct comparison of the material parameters was not available. In the geometric analysis conducted on the five geometries, the mean value of Young’s modulus was 1,512.97 kPa. On the other hand, in the material analysis, the elasticity modules were 516, 708.6, and 1,404.5 kPa for hypo-cellular, cellular, and calcified tissues respectively. The resulting elasticity for fibrotic tissues exceeds the range previously proposed (Caballero et al., 2023). Therefore, the method failed to accurately estimate the stiffness of the lipid tissue, resulting in softer values than the actual ones. However, the maximum principal stress distribution achieved with this method made it possible to obtain approximate values for the stress in the plaque. Supplementary Figures show the qualitative comparison between the actual stress and that resulting from taking into account the modulus of elasticity calculated.

### 3.2 Determination of non-linear properties

**FIGURE 7:**
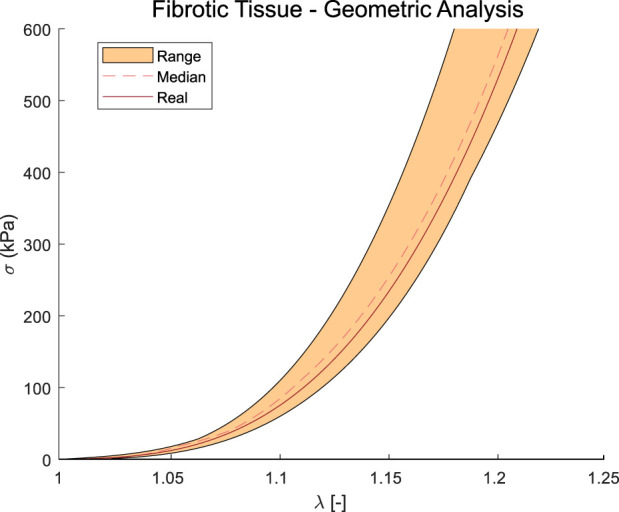
Results of the stress-stretch curves under uniaxial tensile loading obtained with the second approach over the different geometries with the material properties of calcified 1 (Table 2).

In the second approach, we obtained the non-linear properties by using the previous optimization method and adding a Pull-Back algorithm to recover the unpressurized geometry. Since different GOH material parameters (*C*_10_, *k*_1_, *k*_2_, and *κ*) could result in similar curves, we compared the behavior curve rather than the parameter values. Figure 7 displays the median and the range of the resulting behavior curves under uniaxial tensile loading obtained for the different geometries. The error of the estimated curves was computed with the coefficient of determination *R* ^2^, represented in Table 3. Among the analyzed geometries, the first four reached a *R* ^2^ between 0.95 and 0.99. However, more complex geometries, like the fifth plaque, only got a *R* ^2^ of 0.50. Despite this low coefficient, the resulting curves behaved similarly to the real ones. This means that the resulting mechanical response is similar to the actual one.

**TABLE 3:** Summary table to resume the results obtained with the FE models using GOH material models.

	Calcified 1 properties					IVUS 1 geometry			
	IVUS 1	IVUS 2	IVUS 3	IVUS 4	IVUS 5	Calcified 1	Calcified 2	Cellular	Hypocellular
*R* ^2^	0.97	0.99	0.98	0.95	0.50	0.97	0.99	0.88	0.79
*J*_*Linear*_	55.73	72.91	56.35	59.87	55.92	55.73	52.06	23.55	37.39
*J*_*non−linear*_	18.53	33.01	35.30	44.54	44.90	18.53	16.54	16.97	44.75

Five different geometries and four different fibrotic tissues were analyzed. The first data row represents the *R* ^2^ between the real behavior curve and the resulting curve obtained with the second methodology. The last two rows show the cost function value between the $\varepsilon_{rr}^{\mathrm{IV US}}$ and $\varepsilon_{rr}^{\mathrm{iterated}}$ using the first approach (*J*_*linear*_) and the second one (*J*_*non−linear*_).

**FIGURE 8:**
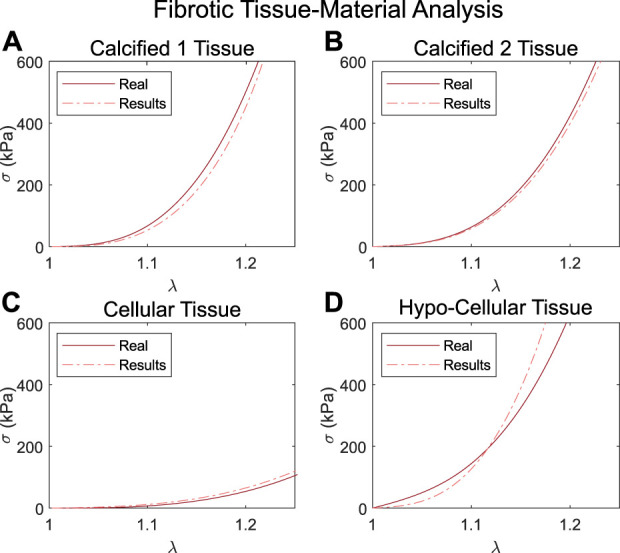
Results of the stress-stretch curves under uniaxial tensile loading obtained with the second approach over the first geometry with the calcified 1 fibrotic tissue **(A)**, calcified 2 tissue **(B)**, cellular tissue **(C)** and hypocellular tissue **(D)**.

For the analysis of different fibrotic tissues, Figure 8 presents the resulting GOH fitted curves under uniaxial tensile loading on the cases with calcified, cellular, and hypocellular fibrotic tissues for the first IVUS geometry. The resulting *R* ^2^ in calcified tissues was 0.97 and 0.99, while for the hypo-cellular and cellular tissues was 0.79 and 0.88.

**FIGURE 9:**
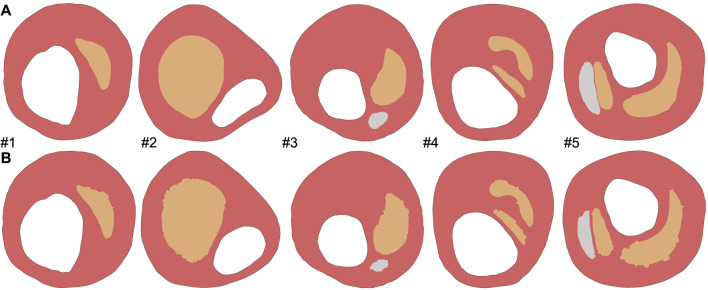
Comparison between the true unpressurized geometries **(A)** with the estimated ZP geometries **(B)**. Fibrotic tissues are represented in reddish color, lipids in orange, and calcifications in gray.

The pattern-search algorithm efficiently minimized the error of the cost function (Eq. 4) in a maximum of 4 h, which is equivalent to about 40 iterations. The final ZP geometries were estimated by using a Pull-Back algorithm optimizing the recovery factor. Figure 9 compares the “true” ZP geometry with the estimated one for different geometries. In most cases, the geometries are similar, except for the second geometry, where the lumen was estimated to be larger than the actual one. Figure 9 shows some differences in the lipid and calcification contours between the unpressurized geometries used to simulate the IVUS data and the unpressurized geometry after the optimization process. The roughness of the contours comes from the error resulting from the segmentation process and the size of the mesh elements. Different SGVs provided different errors and smoother contours (Latorre et al., 2022). The size of the mesh elements was related to the resolution of the IVUS images.

### 3.3 Comparison between approaches

**FIGURE 10:**
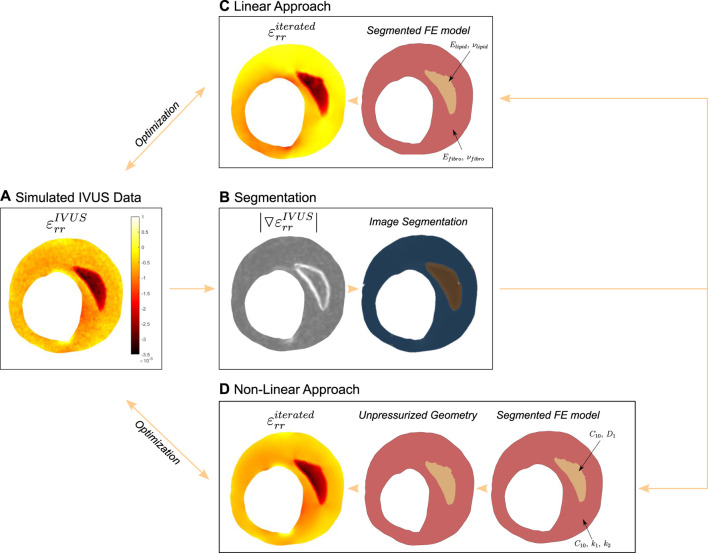
Results of both methods over the first IVUS geometry with calcified 1 material properties. **(A)** Simulated radial strains after adding 20 dB of SNR to the FE results. **(B)** Segmentation process, where the chosen SGV to extract the lipid was $\left|▿\varepsilon_{rr}\right|$ which is represented next to the image segmentation results. Then, the mechanical characterization used this segmentation to estimate the radial strain with linear material properties **(C)** or non-linear properties **(D)**.

Finally, we compared both techniques over the same simulated IVUS data from GOH models. Figure 10A shows the radial strain obtained in the simulated IVUS data, which represents the ground truth in our cost function (Eq. 4). Then, Figure 10B presents the segmentation process, where the SGV was used to get the segmentation of the tissues. Both the simulated IVUS data and the segmentation were the same for the two approaches. We then defined the linear elastic or non-linear hyperelastic material parameters for each tissue, depending on the approach. At the end of each approach, Figures 10C, D present the radial strain maps obtained with the linear and non-linear approaches respectively. On the one hand, the first method used linear elastic materials to mimic a highly hyperelastic behavior, so the resulting cost (*J*_*linear*_) was over 55.73% for the first IVUS geometry. On the other hand, the second one obtained an error in the cost function (*J*_*non−linear*_) of 18.53%. All the cost values are collected in Table 3. As a summary, it can be stated that, in all cases, the second method reduced the mean error in the cost function, providing more accurate radial strain maps.

**FIGURE 11:**
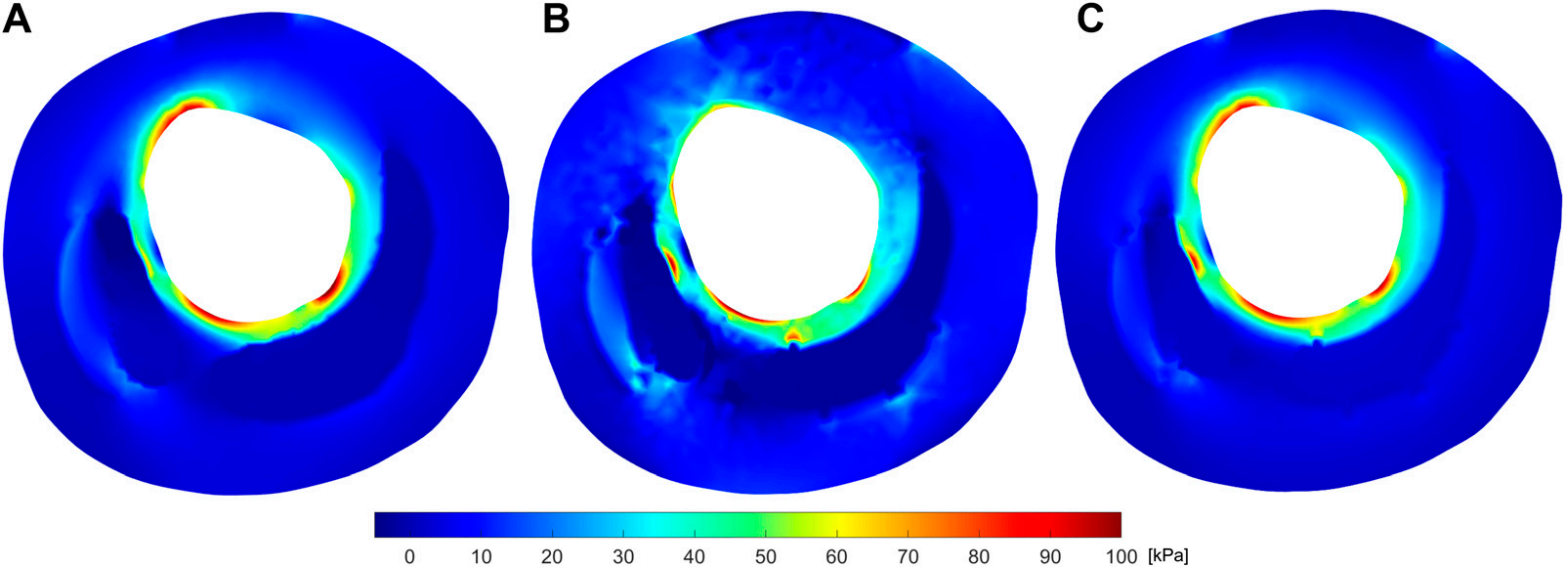
Max. Principal Stress distribution [kPa] at 115 mmHg in the fifth IVUS plaque taken as ground truth **(A)**, and the resulting (*σ*_max_) for the linear **(B)**, and non-linear **(C)** approaches.

Once the material properties and the unpressurized geometry had been estimated, it was possible to calculate the stress distribution on the plaque. Figure 11 shows the maximum principal stress (*σ*_max_) maps resulting from the linear and non-linear approaches compared to the ground truth for the fifth geometry. Although the method gave a low *R* ^2^ for fibrotic tissue in this geometry, the stress distribution in the second approach is more similar to the true one compared to the first approach. Supplementary Figure S1 compares the stresses between the ground truth and the results of both approaches for the other four geometries, and the second figure shows the stress distributions for the different fibrotic tissues. It can be seen that in all cases both approaches were able to reproduce the stress distribution and the areas of maximum values. However, the results suggested that the second method more accurately reproduced the areas of highest stress.

## 4 Discussion

In this study, we compared two different approaches to determine the mechanical properties of atherosclerotic tissues. In the first one, we obtained Young’s modulus of the tissues at a specific blood pressure. This kind of process allowed us to compare the stiffness of different tissues and classify them according to their behavior. If we were able to capture images of the plaque over time, it could also be helpful in evaluating the evolution of the pathology and the result of some treatments. However, atherosclerotic tissues exhibited a significant non-linear behavior (Narayanan et al., 2021; Torun et al., 2022). Thus, this method provided only a relative Young’s modulus that did not fully explain the behavior of the tissue. To overcome this limitation, we proposed the second approach, which consisted of the use of a Pull-Back algorithm to recover the unpressurized geometry, we tried to characterize the full non-linear behavior of atherosclerotic plaque tissues. The process yielded an estimation of the mechanical properties and ZP geometry at the same time, which enabled the determination of the stress state over the atherosclerotic plaque under physiological conditions.

### 4.1 Determination of linear elastic properties

To assess the robustness of the method, we initially simulated IVUS data using FE models with Neo Hookean materials. This procedure estimated Young’s modulus of the tissues, which was then compared with the Neo Hookean parameters using the relationships outlined in Eqs 2, 3. Although Neo Hookean models were simple for describing the behavior of the arterial tissues, this type of model has been considered enough for capturing the mechanical response of the plaque (Akyildiz et al., 2016; Noble et al., 2020). The geometries analyzed in this theoretical study were previously used in other *in silico* works, where the FE models were built directly with linear elastic properties and a lumen pressure of 1 kPa (Le Floc’h et al., 2009; Bouvier et al., 2013; Tacheau et al., 2016). These studies did not consider the hyperelasticity behavior of the tissues and obtained a direct correlation between the results and the Young’s modulus used in their FE models. Our approach successfully characterized lipid and fibrotic tissues in different geometries and material combinations with similar results as those presented in literature (Le Floc’h et al., 2009; Bouvier et al., 2013). Results suggested that *sr* was higher for the fibrotic than the lipidic tissues. This was because the *sr* depended on the relative error of the Young’s modulus, so even if the estimated value for the lipid was 12 kPa instead of the actual value of 10 kPa, it could still result in an *sr* of 80%. Calcifications were estimated as highly stiff solids, with two orders of magnitude above the other tissue. However, the estimated values were far from the actual ones. Similar outcomes were reported by Le Floc’h et al. (2009) and Tacheau et al. (2016) who successfully identified calcium inclusions but failed to accurately estimate their Young’s modulus due to the small strains amplitudes. Nevertheless, the differences between considering the actual or the estimated Young’s modulus did not affect the radial strains map.

In addition to the geometric influence, we also analyzed the impact of the materials on the mechanical characterization. It should be noted that material combinations between lipid and fibrotic tissues played a key role in Young’s modulus estimation. For atherosclerotic plaques with softer fibrotic tissues, the methodology yielded worse mechanical characterization and, in those cases, the stiffness of the lipid seemed to have no influence. The segmentation process, which was based on SGV, was also found to be slightly affected by the material combination (Latorre et al., 2022). Although cases with less gradient between the stiffness of the lipid and fibrotic tissues were more challenging for segmentation (Latorre et al., 2022), the segmentation was performed properly in all cases. It is worth noting that the cases that were more challenging for segmentation were not the same as those with worse *sr*. Figure 5 shows that the mechanical characterization was more dependent on the geometry rather than the atherosclerotic materials. The optimization procedure was conducted by using a pattern-search algorithm instead of a gradient-based method, as *fmincon* algorithm, used in previous studies (Le Floc’h et al., 2009). The newly chosen algorithm provided faster results and showed less dependence on the initial point in the optimization process.

After the validation with Neo Hooke models, we applied the methodology to more realistic FE models with fibrotic tissues modeled as GOH material in order to reproduce real arterial tissue behavior. In these cases, it was not possible to directly compare the estimated Young’s modulus with the GOH material parameters. However, stiffness values were found to be over the limits (Caballero et al., 2023). Due to the high Young’s modulus estimation of fibrotic tissues, lipids appeared to be softer than their actual stiffness values. This overestimation of the fibrotic tissue stiffness was the result of trying to describe a highly non-linear hyperelastic material with a linear elastic model. As can be seen in Figure 11, and in the Supplementary Figures, the maximum principal stress field obtained with linear properties had some similarities with the true field. However, the properties obtained are unrealistic and the stresses are only close to the study pressure and cannot be generalized to other physiological pressures. This was because the estimated Young’s modulus was obtained as the slope of the straight line secant to the real curve. As a result, the method provided a relative stiffness value at a certain blood pressure, which could assist in determining the nature of the tissues but would not provide their actual behavior. We applied the process at different pressure loads (80–85, 110–115, and 135–140 mmHg) and observed an increase in the relative stiffness at higher pressures, although these results were not included in the current paper. Akyildiz et al. (2016) used a similar process to obtain the stiffness of the plaque tissues at systolic and diastolic pressures, proving the same outcome that stiffness increases over the cardiac cycle.

### 4.2 Determination of non-linear properties

In this method, due to the highly non-linearity of the problem, a Pull-back algorithm was included to compute the ZP geometry in order to obtain a better estimation of the hyperelastic material parameters. A correct reference geometry was important not only for the stress distribution but also for the estimated vessel diameter (Alastrué et al., 2008). The first approach used a reference geometry of the pressurized state at 110 mmHg, assuming a linear elastic behavior of the tissues. Thus, this pressurized reference could lead to Young’s modulus acquisition (Le Floc’h et al., 2009; Bouvier et al., 2013; Tacheau et al., 2016) or orthotropic linear properties (Gómez et al., 2019). However, this assumption was not valid for estimating non-linear materials, such as soft tissues. In order to consider the unpressurized geometry, some studies obtained the Yeoh material parameters by taking *ex-vivo* images or tests of the atherosclerotic plaque (Akyildiz et al., 2016; Torun et al., 2022) or using the pressurized geometry at low pressures as ZP geometry (Narayanan et al., 2021), which could lead to a more rigid characterization. In the present paper we implemented a modified version of a Pull-Back algorithm defined by Raghavan et al. (2006), to estimate an initial unpressurized geometry of aneurysms avoiding the iterative process by fixing the recovery factor *K*_*ZP*_ = 1. Although the estimated initial geometry was not entirely accurate, it was continually updated with each material evaluation. At the end of the iterative process, we got the hyperelastic material parameters for the atherosclerotic tissues. Subsequently, a more accurate initial geometry was obtained by optimization of the Pull-Back algorithm by fixing the mechanical properties without constraining the recovery factor. For both approaches, we used an i7-10700K CPU with 8 cores running at 3.79 GHz and with 64 GB RAM. While the first approach took around 2 h to accomplish around 180 evaluations, the second approach needed twice time to get about 40 iterations. This was due to the complexity of the second approach which included the Pull-Back step and the convergence velocity of the non-linear tissues.

This new technique was successfully applied to five different geometries and four different fibrotic materials (cellular, hypocellular, and two calcified). Results showed that different GOH material parameters lead to similar curves and in all analyzed cases the fibrotic tissues were correctly characterized. The resulting *R* ^2^ was above 0.95 showing similar behavior curves to the real ones, except for complex geometries or hypo-cellular tissues. Complex geometries, like the fifth IVUS, had a worse estimation of the curve, due to the presence of two lipids and one calcification that shielded the strain maps in those regions. Moreover, other fibrotic tissues were more challenging to estimate during the optimization process. We analyzed many different initial points for the pattern-search algorithm with similar outcomes and results suggested that fibrotic materials were considered slightly stiffer than the actual ones. This was the result of assuming the recovery factor fixed *K*_*ZP*_ = 1 during the optimization. Lipid Neo Hookean parameters were obtained with a lot of variation regarding the actual values, especially in complex geometries, where the estimated values were close to the initial point of optimization. For softer fibrotic tissues, such as cellular and hypocellular, the mechanical properties of the lipid played a more important role in estimating the properties of the fibrotic tissue. The stiffness of calcifications was determined with a similar level of error as in the first approach. Therefore, both approaches were consistent with results reported in the literature, indicating that the exact Young’s modulus was difficult to assess due to the small strain variation over a rigid solid (Le Floc’h et al., 2009; Tacheau et al., 2016), as well as the high stiffness of calcifications compared to fibrotic tissues (Gijsen et al., 2021).

Despite the lipid and calcification results, the final strain maps $\left(\varepsilon_{rr}^{\mathrm{estimated}}\right)$ were close to the ground truth, resulting in a low error in the cost function. Torun et al. (2022) computed the mechanical properties of all plaque tissues but they focused mainly on fibrotic tissue. This suggested that strains observed in the evaluated atherosclerotic plaques were mainly influenced by the fibrotic tissues rather than the lipid core. One advantage of the proposed approach was that, while the majority of the literature used information on the diastolic and systolic pressures for determining the mechanical properties (Liu et al., 2018; Liu et al., 2019); this approach could be applied at any state of pressure. Since the methodology estimates the material parameters of GOH, we obtain the response for all ranges of pressures independently of the pressures used for the estimation of the parameters. So, the results could be extrapolated to other lumen pressures. Once the material properties were finally estimated, we applied the Pull-Back algorithm without fixing the recovery factor to obtain the final unpressurized geometry. With this geometry and the estimated mechanical properties, it would be possible to determine the stress state in the arterial wall and apply it to the risk of rupture of the plaque. Although Figure 11 and Supplementary Figures show that both approaches gave similar stress distributions to the ground truth at 115 mmHg, the second method could be extrapolated to any other physiologic pressure. Furthermore, this approach produced a smoother and more accurate *σ*_max_ distribution in the different analyzed cases even in complex geometries where the optimized material properties provided a low *R* ^2^. Thus, this method would allow a better estimation of the areas with the highest stress and therefore the areas with the highest risk of plaque rupture.

### 4.3 Limitations

Although the results of this new methodology are very promising, it is important to note that the study has a number of significant limitations.

• The study was basically theoretical and should be considered as the first step to lay down a methodology and validate it with different geometries and materials. We used the radial strains in our cost function because it was commonly used in the literature (Le Floc’h et al., 2009; Tacheau et al., 2016; Gómez et al., 2019). However, we also tried to use displacement fields with similar outcomes (Akyildiz et al., 2016). Radial strains or displacement fields could be obtained from IVUS images by using speckle tracking or other algorithms (Maurice et al., 2004; Lopata et al., 2009), that had been successfully applied *in vitro* (Le Floc’h et al., 2010; Porée et al., 2017) and *in vivo* (Le Floc’h et al., 2012). Due to the noisy nature of IVUS data, the radial strains obtained by these estimators lead to noisy strain maps. We mimicked that noise by adding an SNR of 20 dB over the FE strain field.
• The *in silico* data were constructed using 2D FE models under the assumption of plane strain. These FE models overestimated the magnitude of stress compared to 3D FE models (Ohayon et al., 2005; Carpenter et al., 2020; Peña et al., 2021). Narayanan et al. (2021) developed a method to create meshes from OCT images, and they captured not only the 3D morphological information but also ensured that the applied load was physiologically representative. This kind of process needed different segmented slices over the axial direction of the artery which would increase the segmentation error in complex geometries. We used 2D FE models to simulate the information provided by a standard IVUS image. Furthermore, the influence of residual stress was not taken into account in this study. Although it has a relevant impact on the location of the maximum stress in the atherosclerotic plaque (Cilla et al., 2012), residual stress requires *ex vivo* information that is difficult to obtain (opening angle test, axial stretch…).
• The fibrotic tissues were considered homogeneous, with the same mechanical properties as the tissue. Nonetheless, histologies showed a heterogeneous composition, and it affected the mechanical properties and the stress state (Akyildiz et al., 2016). However, heterogeneities were very difficult to segment or detect and some methodologies took them into account by changing the number of inclusions evaluated in their models and obtaining heterogeneous Young’s modulus over the fibrotic tissue (Le Floc’h et al., 2009; Porée et al., 2017). In this study, only fibrotic tissue and macro inclusions, such as lipids or calcifications, were segmented, and homogeneous behavior was assumed in each tissue.
• The optimization process was set to take no more than 4 h; but it depended on the initial point and the limits of every parameter. A previous study was conducted to analyze different initial points with similar results. Although pattern-search was a local optimization algorithm, the polling method was modified to avoid local minima. We also compared these results with those provided by the genetic algorithm, but the required time was much longer. More complex global optimization methodologies, like modifications of Bayesian optimization (Torun and Swaminathan, 2019), take more time (approximately 7 h) to estimate the hyperelastic material properties of the arterial wall (Torun et al., 2022). Liu et al. (2019) managed a computational cost of 1–2 h for determining the mechanical properties of ascending thoracic aortic aneurysm. They reduced the time by using principal components analysis from the stress-stretch curves and using an algorithm to go from coarse to fine to analyze lots of behavior curves in less time (Liu et al., 2018; Liu et al., 2019). They characterized the properties assuming the arterial wall with the same properties, however, in atherosclerotic plaques, the importance remained on the different properties of the tissues, and this method should be applied for each segmented material increasing the computational cost and the complexity of the study.

### 4.4 Conclusion

In this work, we have presented a new method to determine the non-linear material properties of atherosclerotic tissues. The proposed approach had been compared with a classical process based on linear properties, providing a more accurate description of the mechanical behavior of atherosclerotic tissues and resulting in a lower error in the cost function. We estimated the non-linear properties of the tissues and the unpressurized geometry of the plaques, which allowed us to obtain the mechanical response of the atherosclerotic tissues throughout the entire cardiac cycle, rather than only at a specific blood pressure. Despite being a theoretical framework, this method was successfully applied to different real geometries and different fibrotic materials, demonstrating its potential as a valuable tool for assessing the vulnerability of patients with atherosclerotic coronary plaques.

## Data Availability

The original contributions presented in the study are included in the article/Supplementary Material, further inquiries can be directed to the corresponding author.
