# Supplementary material for: Characterizing atherosclerotic tissues: in silico analysis of mechanical properties using intravascular ultrasound and inverse finite element methods
Source: Front Bioeng Biotechnol. 2023 Dec 13;11:1304278. doi: 10.3389/fbioe.2023.1304278 (PMC10751321; doi:10.3389/fbioe.2023.1304278)
Supplement: Supplementary file 1 [file DataSheet1.PDF]

Supplementary Material

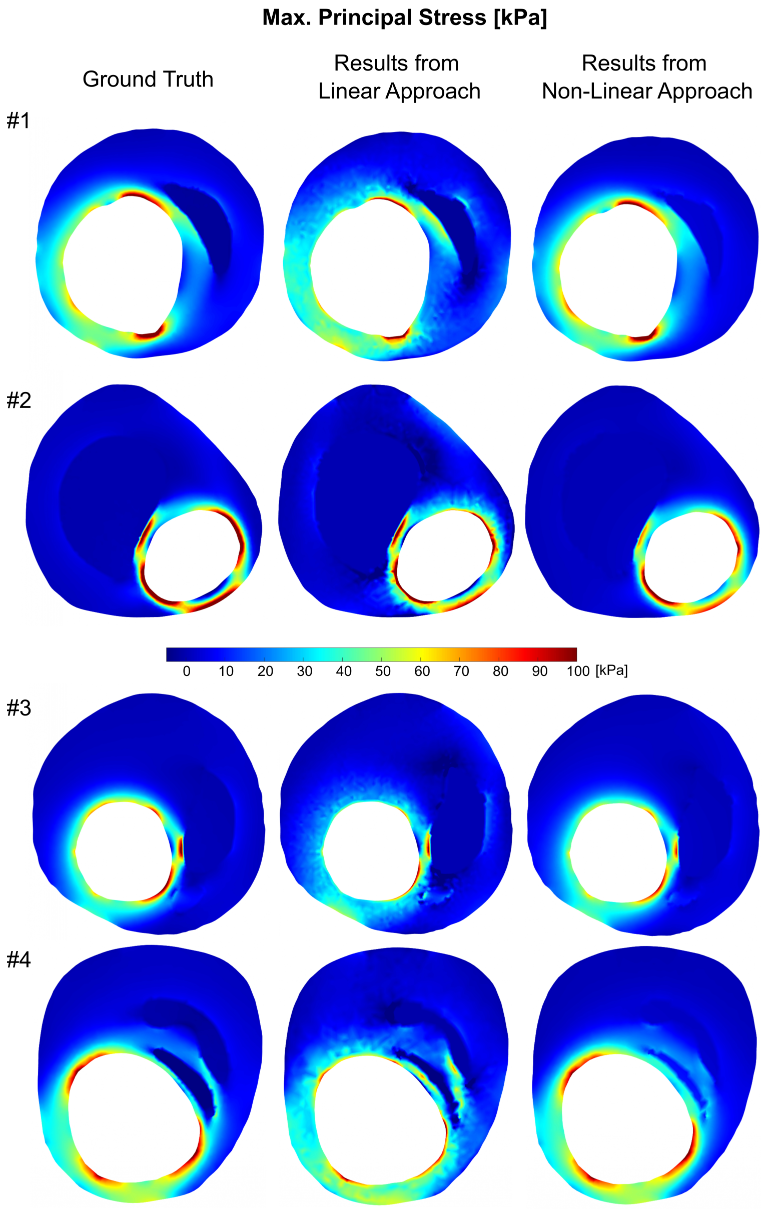

Figure S1. Max. Principal Stress distribution [kPa] at 115 mmHg in four different geometric plaques, all simulated with the *calcified 1* tissue. Comparison between ground truth (σ_{max}) (first row), results of the linear approach (second row), and non-linear approach (third row).

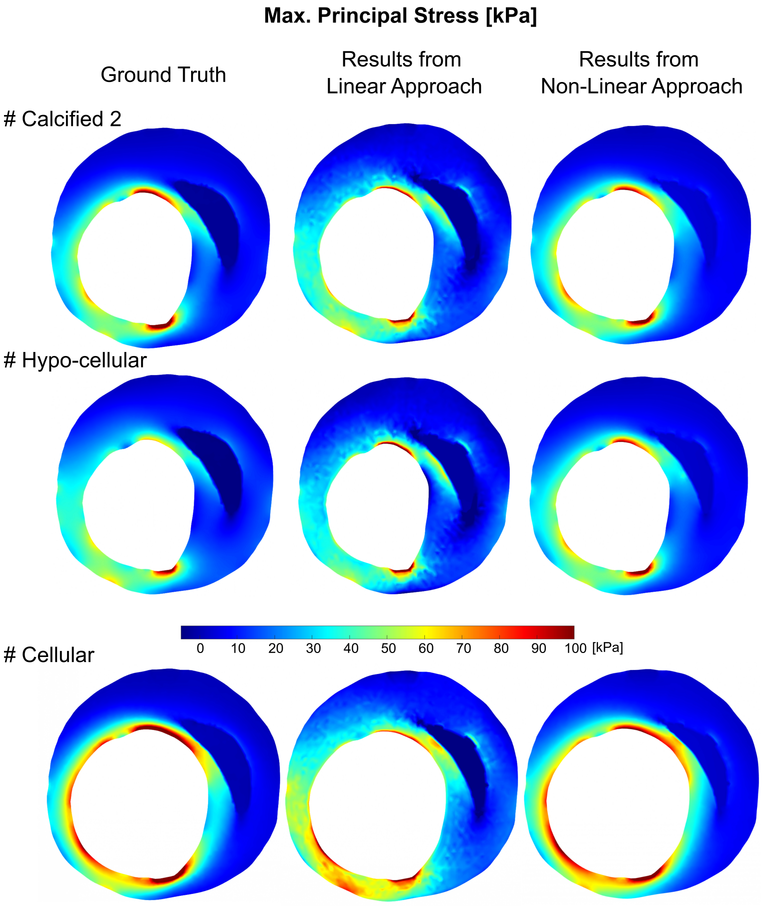

Figure S2. Max. Principal Stress distribution [kPa] at 115 mmHg in the first IVUS geometry with different fibrotic tissues (calcified, hypo-cellular, and cellular). Comparison between ground truth (σ_{max}) (first row), results of the linear approach (second row), and non-linear approach (third row).
